# Parameter optimization in HN‐IMRT for Elekta linacs

**DOI:** 10.1120/jacmp.v10i2.2951

**Published:** 2009-04-28

**Authors:** Danielle Worthy, Qiuwen Wu

**Affiliations:** ^1^ Department of Radiation Oncology Wayne State University Detroit Michigan 48201 USA; ^2^ Department of Radiation Oncology William Beaumont Hospital Royal Oak Michigan 48073 USA

**Keywords:** IMRT, DMPO, head‐and‐neck, delivery efficiency, dosimetric accuracy

## Abstract

Planning and delivery in HN‐IMRT has been challenging for the Elekta linac because of numerous machine limitations. Direct aperture optimization (DAO) algorithms have had success in simplifying the planning process and improving plan quality. Commercial adaptations of DAO allow for widespread use in many clinics; however clinical validation of these methods is still needed. In this work we evaluated Pinnacle^3^ commercial software for HN‐IMRT on the Elekta linac. The purpose was to find a set of planning parameters that are applicable to most patients and optimal in terms of plan quality, delivery efficiency, and dosimetric accuracy. Four types of plans were created for each of 12 patients: ideal fluence optimization (FO), conventional two‐step optimization (TS), segment weight optimization (SW), and direct machine parameter optimization (DMPO). Maximum number of segments (NS) and minimum segment area (MSA) were varied in DMPO. Results showed DMPO plans have the best optimization scores and dosimetric indices, and the most consistent IMRT output among patients. At larger NS (≥80), plan quality decreases with increasing MSA as expected, except for $\text{MSA}<8\;{\text{cm}}^{2}$, suggesting presence of local minima in DMPO. Segment area and MUs can vary significantly between optimization methods and parameter settings; however, the quantity ‘integral MU’ remains constant. Irradiation time is linearly proportional to total plan segments, weakly dependent on MUs and independent of MSA. Dosimetric accuracy is independent of DMPO parameters. The superior quality of DMPO makes it the choice for HN‐IMRT on Elekta linacs and its consistency allows development of ‘class solutions’. However, planners should be aware of the local minima issue when pushing parameters to the limit such as NS<80 and $\text{MSA}<8\;{\text{cm}}^{2}$. The optimal set of parameters should be chosen to balance plan quality and delivery efficiency based on a systematic evaluation of the planning technique and system constraints.

PACS number: PACS: 87.55.D, 87.55.de

## I. INTRODUCTION

Intensity‐Modulated Radiotherapy (IMRT) has become a standard delivery technique in clinics across the US and worldwide.^(1)^ Conventionally, IMRT plans are created in two steps: 1) fluence map (beamlet) optimization, followed by 2) multileaf collimator (MLC) conversion where optimized fluence profiles are decomposed into a set of deliverable MLC segments using leaf sequencing algorithms.^(^ ^2^ ^,^ ^3^ ^)^ One main criticism of two‐step IMRT optimization is that the planning process and deliverable plans are unnecessarily complex. This is commonly observed in the treatment of head‐and‐neck cancer, where planning is complicated due to the close proximity of critical structures to large and irregular target volumes.^(^ ^4^ ^,^ ^5^ ^)^ Additionally, in simultaneous integrated boost (SIB) HN‐IMRT plans, the primary tumor and subclinical disease are concurrently treated with one treatment plan. This allows for concurrent dose escalation to the primary tumor, requiring multiple prescription dose levels in the plan.^(6)^ This increases the size of target volumes as well as differences between prescription and critical structure tolerance doses, making planning more difficult. Greater plan complexity increases the total number of segments in the deliverable plan (some of which may be too small to be delivered accurately), as well as the total monitor units (MUs).^(^ ^2^ ^,^ ^7^ ^–^ ^10^ ^)^ This prolongs patient treatment times and decreases delivery efficiency, patient throughput and delivery accuracy, as patient movement during treatment is less avoidable.^(11)^ ^,^ ^(12)^

The increased complexity of deliverable IMRT plans stem from the fact that constraints on the beam delivery systems (MLCs and diaphragms) are accounted for separately during MLC conversion and not within the optimization process itself.^(8)^ ^,^ ^(13)^ Furthermore, by realizing delivery limitations in a separate leaf sequencing process, significant deviations between the deliverable and optimal dose distributions are observed. This lengthens treatment planning time because empirical adjustment of the IMRT objective function parameters and reoptimization are often required.^(3)^ ^,^ ^(14)^

In addition to treatment site location, resulting plan complexity and deliverable plan degradation will also depend on other factors, such as the delivery system limitations. Planning and delivery are especially challenging for Elekta linacs because of numerous constraints on the beam delivery systems, some of which include: a) inability of the X‐diaphragms to cross the central axis,^(7)^ ^,^ ^(15)^ b) a minimum of 1 cm gap between opposing leaves and, more importantly, 1 cm gap between leaves adjacent to the opposing leaf,^(16)^ and c) to a lesser degree, a limited over travel distance of 12.5 cm for MLC leaves and Y‐diaphragms.^(17)^

These Elekta machine constraints have undesirable effects on the planning process and deliverable treatment plan, some of which are described here.

**Figure 1:**
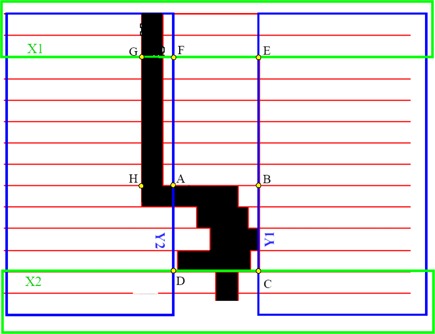
Schematic of the ‘flagpole’ effect for Elekta MLC. X‐diaphragms are outlined in green and backup Y‐diaphragms in blue. X1‐diaphragm stops at the central axis denoted by point G. MLC leafs are outlined in red.

First, the combination of constraints (a and b above) causes ‘flagpole’ effects for small off‐axis segments. These are frequently found in HN‐IMRT plans due to a large separation between the isocenter (neck region) and areas of higher dose (head region), as well as large differences between prescription and tolerance doses. ‘Flagpole’ effects occur because the X‐diaphragms are unable to fully collimate the field in addition to the MLC leaves, resulting in unwanted radiation to regions outside of targets.^(15)^ ^,^ ^(18)^ A technique to reduce unwanted radiation is illustrated in Fig. 1. The area to be treated is outlined by rectangle ABCD, which is located off the central axis. The X1‐diaphragm stops at the central axis G, such that the unwanted radiation is largely blocked by the MLC and backup Y‐diaphragms. In this approach, the MLC leaves are advanced past the open segment area (ABCD), so that the backup Y‐diaphragm can shield the minimum gap (area AFGH) between the opposing MLC leaves.^(15)^ The Y‐diaphragms are much thinner than the X‐diaphragms, having a thickness of only 3 cm.^(16)^ Thus, the transmission through the minimum gap (AFGH) is ~10%, significantly larger than the 2% transmission through the MLC leaves alone (area ABEF) and the 0.5% transmission through the X‐diaphragms.^(19)^ Second, constraint (b) prevents MLC leaf inter‐digitization, and the 1 cm minimum gap requirement makes leaf sequencing and optimization of segment shapes very difficult. This is because any change in the position of a given MLC leaf can potentially affect the position of all MLC leaves in the same leaf bank and opposing leaf bank, completely altering the segment shape. This severely restricts segment shape possibilities for the leaf sequencer or optimizer. Third, constraint (c) also restricts segment shape possibilities by limiting leaf over travel distance, but to a much lesser extent in comparison to constraint (b). All of these adverse effects caused by the Elekta machine limitations lead to very complex HN‐IMRT plans with large differences observed between optimized and deliverable dose distributions.

To address the limitations of two‐step optimization, researchers have developed aperture‐based or segment‐based optimization, a method where beam delivery system constraints are accounted for during optimization. With this approach, the shapes and weights of the MLC segments are optimized simultaneously, leading to the production of a deliverable plan without the use of a separate leaf sequencing procedure.^(^ ^8^ ^,^ ^9^ ^,^ ^14^ ^,^ ^20^ ^–^ ^22^ ^)^ Therefore, deliverable dose distribution degradation is prevented, and further reoptimization is not required.^(7)^ ^,^ ^(21)^ ^,^ ^(23)^ Another advantage of aperture‐based optimization is its ability to grant the treatment planner direct control of IMRT plan complexity by introducing additional planning parameters such as the total number of plan segments within the optimization process.^(8)^ ^,^ ^(9)^ ^,^ ^(21)^ A number of aperture‐based optimization algorithms have been published^(^ ^8^ ^,^ ^9^ ^,^ ^13^ ^,^ ^14^ ^,^ ^20^ ^,^ ^24^ ^)^ and compared with the conventional two‐step method. Published results have shown that aperture‐based optimization creates simpler treatment plans with fewer segments and fewer monitor units while still maintaining similar or better plan quality, as compared with conventionally optimized plans.^(^ ^2^ ^,^ ^3^ ^,^ ^8^ ^–^ ^10^ ^,^ ^20^ ^,^ ^23^ ^–^ ^25^ ^)^

There are many benefits of plan simplification such as reduced patient treatment times and improved delivery efficiency. The success of aperture‐based algorithms in achieving plan simplification raises the following questions: 1) To what extent can treatment plans be simplified without significant sacrifices in plan quality? and 2) What parameter settings are optimal for different treatment sites? Currently, there are very few published studies that have systematically searched for the answers to these questions. Additionally, most of the studies previously conducted to compare aperture‐based optimization with conventional methods have done so in a research environment, using outdated commercial software and in‐house built aperture‐based algorithms. Clinical users do not have access to the same resources as researchers when working with an aperture‐based algorithm that has been adapted for commercial use. Therefore, they cannot implement algorithms in the same manner. This makes translation of published findings to the everyday clinic difficult and makes the results unpredictable, necessitating clinical validation of these optimization methods.

In 2008, Ludlum and Xia^(2)^ and Jones and Williams^(3)^ performed studies that compared direct machine parameter optimization (DMPO), a commercial adaptation of aperture‐based optimization available within the Pinnacle^3^ treatment planning system, to conventional two‐step optimization for Siemens and Varian linacs. For Siemens linacs, Ludlum and Xia found that DMPO is a practical and preferable alternative to conventional optimization for prostate and head‐and‐neck planning. With regard to parameters, they recommended a minimum of 40 segments for prostate plans and 50 segments for head‐and‐neck plans. For Varian linacs, Jones and Williams observed considerable advantages in the dosimetric quality of DMPO plans and, for HN‐IMRT plans with 6–9 beams, determined that a value of 14 segments per beam was optimal. Comparatively, Elekta linacs have more restrictions on the beam delivery systems than both Siemens and Varian linacs and therefore these findings are expected to differ significantly.

In this work, using commercially available treatment planning software, we systematically evaluated planning parameters for HN‐IMRT on an Elekta linac with three specific goals. First, we aimed to find the best IMRT optimization method available in the Pinnacle^3^ treatment planning system and used it as a benchmark. Optimization methods that were compared include ideal fluence optimization, conventional two‐step optimization, segment weight optimization, and DMPO. Second, we aimed to determine the effects that planning parameters specific to DMPO have on plan quality. Last, we aimed to find a set of planning parameters that are applicable to most patients and optimal, not only in terms of plan quality as has been done in other studies, but of delivery efficiency and dosimetric accuracy as well. We have proposed a new concept, ‘integral MU’, to explain the relationship of MUs and segment area for different types of plans.

## II. MATERIALS AND METHODS

### A. Patient data and Treatment Planning Parameters

Twelve SIB HN‐IMRT cases were randomly selected for this study, with plans created on the Pinnacle^3^ treatment planning system (Pinnacle^3^ v7.4f, Phillips Radiation Oncology Systems, Madison, WI). For all plans, 7 coplanar 6 MV photon beams were evenly distributed around the neck of the patient. Some of the volumes of interest used in SIB HN‐IMRT planning are briefly described here. The gross tumor volume, GTV1 encompassed the primary tumor and involved lymph nodes (>1 cm); a 5 mm margin was used for expansion to the clinical target volume, CTV1. A second clinical target volume, CTV2, included all lymph nodes that are at potential risk and need elective treatment. A 5 mm margin was used for expansion from each CTV to its respective planning target volume, PTV. Critical structures included the brainstem, spinal cord, mandible and parotid glands. For the brainstem and spinal cord, an additional safety margin was used to create appropriate planning risk volumes (brainstem+3 mm and cord+5 mm).

Dose and dose–volume‐based optimization objectives for targets and organs‐at‐risk (OARs) were used in the planning for all patients. For each objective, the planner can set a target dose as well as a weight or penalty factor.^(26)^ These parameters (target dose and weight) were iteratively adjusted in a trial‐and‐error fashion until a standard set of parameters was found to yield optimal plan quality for most patients (see Table 1). In Table 1, the region of interest (ROI) ring represents normal tissues not accounted for by any other objectives and therefore excludes all targets and OARs. The purpose of this ROI is to limit dose to normal tissues and effectively remove hot spots located outside of target volumes.

**Table 1:** Optimal set of objective function parameters that were used for all plans for each patient.

*ROI*	*Type*	*Dose (Gy)*	*% Volume*	*Weight*
PTV1 no mandible	Uniform dose	73	—	50
PTV1 no mandible	Min DVH	73	95	1
PTV2 alone	Uniform dose	62	—	40
Rt Parotid alone	Max DVH	20	60	1
Rt Parotid alone	Max DVH	30	20	1
Lt Parotid alone	Max DVH	20	60	1
Lt Parotid alone	Max DVH	30	20	1
Cord	Max dose	38	—	10
Cord+5 mm	Max dose	43	—	50
Brainstem+3 mm	Max dose	47	—	10
Mandible	Max dose	65	—	75
Ring	Max dose	47	—	2

### B. comparison of optimization Methods

**Figure 2:**
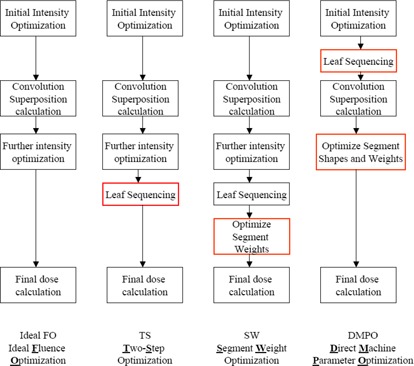
Flow chart summarizing four optimization techniques available in Pinnacle^3^ that were compared in this study. Reading left to right: boxes highlighted in red represent the differences between each specific optimization type and the previous listed method. This modified from van Asselen et al.^(22)^ flow diagram.

The Pinnacle^3^ treatment planning system uses a gradient search method for optimization and is capable of four optimization methods: ideal fluence optimization (FO), conventional two‐step optimization (TS), segment weight optimization (SW), and direct machine parameter optimization (DMPO). The differences between these four optimization methods are summarized in Fig. 2. In ideal FO, the optimizer generates a fluence map (beamlet) or opening density matrix (ODM) for each beam and the value at each pixel is adjusted iteratively to minimize the cost or objective function value. In TS optimization, ideal FO is followed by MLC conversion to create a deliverable plan. Strictly speaking, MLC conversion is not part of the optimization process since it does not continue to decrease the value of the objective function.^(2)^ For this study, the chosen leaf sequencer used was the k‐means clustering algorithm available in the planning system. In SW optimization, the TS process is taken one step further by optimizing the weight or MU associated with each segment, i.e. post conversion optimization. A detailed description of the DMPO algorithm has been discussed elsewhere.^(7)^ ^,^ ^(27)^ In general, DMPO is similar to SW optimization, but goes a step further as it optimizes the MLC leaf positions simultaneously with the segment weight, making adjustments to both in order to minimize the value of the objective function. With DMPO, there are additional parameters that can be defined by the treatment planner. These include the maximum number of segments (NS), the minimum segment area (MSA), and the minimum number of MUs per segment allowed for the plan. In order to evaluate the different optimization methods, four types of plans were created (one for each optimization technique) and compared for all patients. For DMPO plans, the following parameter settings were used: $\text{NS}=80,\text{MSA}=8\;{\text{cm}}^{2}$, and minimum monitor units per segment setting of 2 MU.

### C. Effect of DMPO Planning Parameters on Plan Quality

In addition to studying the differences in plan quality achieved using each of the optimization methods, it was desirable to determine the effect that the DMPO planning parameters, NS, and MSA have on plan quality. For each of the twelve patients, plans were created with varying NS and MSA while the minimum MU per segment was kept constant at a value of 2. The NS was varied between 20–320 and the MSA parameter was adjusted between 2 cm^2^ and 32 cm^2^, representing typical ranges that were used in the clinic. The plans were compared for all patients.

### D. Plan Evaluation

Treatment plans were evaluated based on plan quality metrics, number of plan monitor units (MUs), and number of actual segments created $\left({\text{NS}}_{\text{actual}}\right)$. Plan quality was evaluated based on the plan score, dose distributions, and selected dosimetric indices for the targets and OARs. The plan score or cost function value takes into account the subscore for each optimization objective or constraint. If an optimization objective or constraint is met, then the subscore is zero. The objectives are all based on dose or dose‐volume and are in the form of quadratic dose differences; therefore, the lower the composite score is, the better the quality of the treatment plan. The score is normalized by the total number of voxels. Therefore it is relative and can be compared from patient to patient.

For dosimetric indices, the main planning goals for HN‐IMRT plans are to: 1) achieve $V_{\text{Rx}}$ values ≥90% for targets, where $V_{\text{Rx}}$ represents the percent of target volume receiving the prescription dose in 35 fractions (PTV1 Rx: 70 Gy, PTV2 Rx: 60 Gy); 2) to keep $D_{1}$ values less than specified tolerances (45 Gy for spinal cord, 54 Gy for brainstem, etc.) for OARs, where the $D_{1}$ value represents the dose delivered to 1% of the OAR volume, equivalent to the maximum dose; and 3) to minimize doses to the parotid glands.

### E. Delivery Efficiency

The delivery efficiency was evaluated in terms of the beam‐on time, effective dose rate $\left({\text{DR}}_{\text{eff}}\right)$, and beam‐on efficiency for a set of DMPO plans with varying NS and MSA settings. Some investigators were able to estimate the delivery time for Siemens machines without actual irradiation.^(2)^ ^,^ ^(16)^ ^,^ ^(28)^ However, that would not be a trivial task for Elekta linacs due to the complexity of IMRT delivery, such as the motion of both MLC and diaphragms. Instead, we performed direct measurement of the delivery time with a stopwatch for plans from three patients on an Elekta Synergy linear accelerator. Treatment plans were delivered in quality assurance (QA) mode using the MOSAIQ record and verify system (IMPAC Medical Systems, Sunyvale, CA) with all beams delivered at gantry angle 0°. This means that the beam‐on times measured include irradiation time only and do not account for any extra time between beams (i.e. gantry rotation time, setup time for next beam, etc.). Clinically, it is also important to be able to estimate the total fraction time for treatments with varying DMPO parameters. The total fraction time includes the beam‐on time as well as additional time for patient setup, cone beam CT scan (optional), gantry rotations between beams, and patient removal. Time estimates for each of these steps are summarized in Table 2, where a conservative estimate for the total additional time is ~17 minutes.

**Table 2:** Fraction time estimates.

*Step*	*Time Estimate*
Patient immobilization and setup	2–5 min
Cone Beam CT scan^*^	2–3 min
Gantry Rotation and Positioning for Next Beam	~40 s per rotation (~4 min for 7 beams)
Patient Removal	2–5 min
Total Additional Time (conservative estimate)	~17 min

Outline of steps included in the total fraction time in addition to the beam‐on time and estimates of the time needed for each step. Total additional time for all steps is conservatively estimated at 17 minutes. ^*^CBCT time is for scan only. No online correction is done for HN‐IMRT patients in our clinic.

The average time between beams for gantry rotation and positioning used in the clinic was estimated by retrospectively looking at time stamps from portal images recorded in the iVIEW electronic portal imaging system (Elekta, Crawley, UK). During each fraction, a portal image is taken automatically during the first segment of every beam. The total time between 7 beams, $t_{g}$ can be estimated by:

(1) $$t_{g}=(ts_{b7}-ts_{b1})-t_{b.o..}^{1-6}$$

where ${\text{ts}}_{b7}$ and ${\text{ts}}_{b1}$ are the time stamps for portal images taken at the start of beam 7 and beam 1, and $t_{b,o}^{1-6}$, is the measured total beam‐on time for beams 1 to 6. Data was collected for four HN‐IMRT patients (four fractions each) and averaged, resulting in a mean time between beams of ~40 seconds (~40 minutes for 7 beams).

Effective dose rate $\left({\text{DR}}_{\text{eff}}\right)$ and beam‐on efficiency were calculated for each patient DMPO plan and defined as

(2) $$DR_{eff}=\frac{MU}{t_{b.o.}}$$

and

(3) $$Efficiency(\%)=\frac{DR_{eff}}{DR_{norm}}$$

where MU is the plan monitor units, $t_{b.o.}$ is the measured beam‐on time for all beams in minutes, and ${\text{DR}}_{\text{nom}}$ is the nominal dose rate (475 MU/min for Elekta linac). For standard conformal treatment with a single segment, the ${\text{DR}}_{\text{eff}}$ is the same as ${\text{DR}}_{\text{nom}}$ and the efficiency is 100%. The decrease of ${\text{DR}}_{\text{eff}}$ and efficiency for step and shoot IMRT are caused by the beam pause between the segments.

### F. Dosimetric Accuracy

The dose delivery accuracy of the resulting IMRT plans was measured in phantom with MapCHECK (Sun Nuclear Corp., Melbourne, FL). Specifically we investigated the effect of DMPO planning parameters on dosimetric accuracy by varying NS and MSA. Treatment plans were delivered with all beams at gantry 0° in QA mode. Measurements were made with SSD=95 cm (to MapCHECK surface) and 3 cm of solid water buildup, the same conditions used clinically. Results were analyzed for each plan using MapCHECK software v3.02 to determine a passing percentage (criteria: 2 mm DTA, 3% dose difference, 4% threshold) between the measured and reference planar dose distributions.

## III. RESULTS

### A. Comparison of Optimization Methods

**Figure 3:**
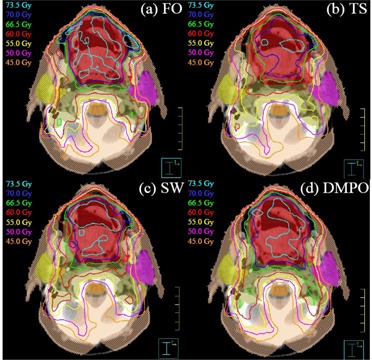
Sample isodose distributions for one patient. Isodose lines: 73.5 Gy (light blue), 70.0 Gy (dark blue), 66.5 Gy (green), 60.0 Gy (red), 55.0 Gy (yellow), 50.0 Gy (magenta), 45.0 Gy (orange). Structures in colorwash: PTV1 (red), PTV2 (brown), brainstem (orange), right parotid (yellow), left parotid (magenta), ring (peach). Isodose lines are least conformal for TS plan and most similar to ideal FO plan for DMPO plan: (a) ideal FO plan, (b) TS plan, (c) SW plan, (d) DMPO plan.

Comparisons of the four different optimization techniques for all twelve patients are summarized in Table 3. As expected, plan quality deviates most from ideal FO plans for TS plans (largest differences in target coverage and plan score) due to the limitations of the diaphragms and MLC. For SW plans, there is a slight improvement in the target coverage and plan score. Among the three deliverable methods, DMPO has the best plan quality (being closest to that of ideal FO plans). This can be seen visually in Fig. 3, which displays an example set of isodose distributions for each of the optimization methods. This figure clearly shows that the isodose lines in the DMPO plan are closest to that of the ideal FO plan with the most conformity to the target volumes (PTV1 and PTV2). To evaluate the statistical significance of the plan quality results for DMPO plans, a paired t‐test was performed. Calculated *p*‐values are shown in the last column of Table 3 where a *p*‐value <0.05 is considered statistically significant. Results show that the improved target coverage and decreased plan scores seen for DMPO plans in comparison to SW plans is statistically significant with all *p*‐values <0.001.

**Table 3:** Optimization methods comparison.

*Evaluation Parameter*	*FO*	*TS*	*SW*	*DMPO*	*p‐value*
PTV1 $V_{\text{Rx}}$ (%)	93.3±2.5	75.9±8.9	80.2±4.2	87.9±3.4	<0.001
PTV2 $V_{\text{Rx}}$ (%)	93.6±3.0	80.2±3.7	80.6±2.5	85.6±3.5	<0.001
Brainstem $D_{1}$ (Gy)	40.3±3.5	41.2±3.9	43.1±4.0	42.1±3.0	0.098
Spinal Cord $D_{1}$ (Gy)	35.1±1.9	37.4±1.7	37.9±1.5	37.4±0.6	0.952
Composite Plan Score	0.078±0.029	0.341±0.102	0.242±0.057	0.150±0.038	<0.001
MU	—–	1007±144	970±148	747±69	<0.001
No. of Segments	—–	—–	76±13	79±1	0.415

Paired t‐tests were performed for values in italics, the pair between DMPO and the best of TS or SW. For all parameters except OAR doses and number of segments, DMPO results are statistically significant (p<0.05).

Regarding critical structures, Table 3 shows very little difference in brainstem and spinal cord maximum doses amongst these techniques, with all values below their tolerances. For the brainstem, paired t‐test analyses (between DMPO and TS) yield a *p*‐value of 0.098, demonstrating that the difference in the maximum doses are not statistically significant. Similarly for the spinal cord, the maximum doses for TS and DMPO were found to be the same, with no statistical significance (p=0.952).

In addition to having the best plan quality, DMPO plans also have the lowest plan MUs, being ~26% less than TS plans and ~23% less than SW plans. The average number of plan segments created for SW and DMPO plans is approximately the same (p=0.415). This was the result of setting proper parameters for both SW and DMPO. However, even though the number of segments created was very similar, the standard deviations were quite different, with DMPO much lower than SW. In fact, for all parameters compared (with the exception of PTV2 $V_{\text{Rx}}$), DMPO has the smallest standard deviations, which demonstrates that consistency in plan quality was achieved.

**Figure 4:**
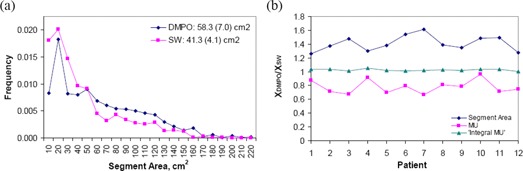
Comparison of DMPO and SW plans. segment area distributions with results averaged over all patients. DMPO creates segments of larger areas compared to that of SW. Legend shows mean segment area and standard deviation (shown in parentheses) for each distribution. parameters (mean segment area, plan MU, and ‘integral MU’) for DMPO plans normalized to SW plans for each patient.

The direct relationship between the number of plan segments and the total MUs required seems intuitive. However, the results from Table 3 show that even though the average number of segments for SW and DMPO plans are approximately the same, the plan MUs are quite different. This indicates that other factors may also influence the total MUs for the plan. A comparison of the segment area distributions for SW and DMPO plans is displayed in Fig. 4(a), with results averaged over all twelve patients. These histograms show that DMPO tends to create plans with larger segment areas $\left(\text{mean}=58.3\;{\text{cm}}^{2}\right)$ compared to the leaf sequencer used to create the segments in TS and SW plans $\left(\text{mean}=41.3\;{\text{cm}}^{2}\right)$. Fig. 4(b) shows the mean segment area, plan monitor units and ‘integral MU’ results for DMPO plans normalized to their respective value for SW plans for each patient. The quantity ‘integral MU’, similar to the concept of integral dose, is defined as:

(4) $$Integral\;MU=\sum_{i=1}^{NS\_actual}SegmentMU_{i}•SegmentAREA_{i}$$

(i.e. the sum of the product of segment MU and segment area for all segments and beams in the plan). In agreement with Fig. 4(a), the mean segment area for DMPO plans is larger than that for SW plans for all patients, with ratios greater than 1. For each patient, the ‘integral MU’ should be approximately constant for each plan created, since it represents the desired spatial dose distribution and depends on the target volumes and objectives. This is validated in Fig. 4(b) where the ‘integral MU’ values for SW and DMPO plans are almost the same, with ratios close to 1. Based on the definition of ‘integral MU’, if the segment area of the plan is larger, as is the case for DMPO plans, then in order for the ‘integral MU’ to be approximately the same as SW, the plan MUs should be less. Fig. 4(b) shows that the plan MUs for DMPO are lower than SW with all ratio values less than 1 also validating the integral MU. This concept explains how DMPO is able to create more delivery efficient plans (i.e. lower MUs) by selectively increasing the area for the chosen segments. However, the approximately equal ‘integral MU’ values between TS, SW and DMPO plans do not prevent small segments with high MUs from being generated in TS or SW plans. This is because for a given small segment with high MU, the product of the segment area and segment MU could be relatively small and therefore would not contribute significantly to the ‘integral MU’, even if the overall plan MUs could be very high. Therefore, if the total MU in TS or SW plans is abnormally higher than other standards plans, one of the possible causes is that there may exist segments with high MUs and small apertures.

### B. Effect of DMPO Parameters on Plan Quality

#### B.1. Maximum Number of Segments

**Figure 5:**
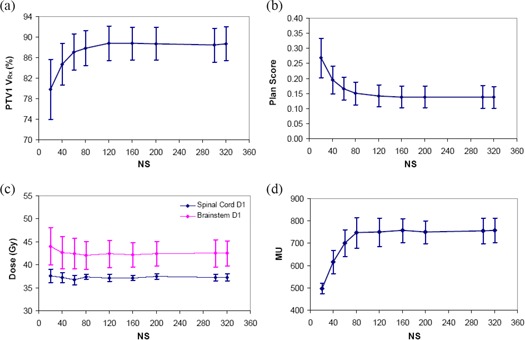
Results of maximum number of segments parameter variation in DMPO study. PTV1 target coverage; (b) plan score; (c) OAR maximum doses; (d) plan MUs. Error bars are one standard deviation.

Figure 5 displays various plan quality metrics and plan MUs as a function of NS setting. As the NS is increased from 20 to 160, the plan quality in terms of target coverage (Fig. 5(a) and plan score (Fig. 5(b) improves noticeably. PTV1 target volume coverage increases by ~10% with similar results for PTV2 coverage (not shown), and plan score decreases by ~48%. The improvement slows down for NS>80. For NS>160, there is no significant improvement in either target volume coverage or plan score; therefore, no clinical benefit is gained in creating plans with NS>160.

The maximum doses of critical structures (brainstem and spinal cord), presented in Fig. 5(c), are relatively independent of the NS setting chosen. The highest $D_{1}$ values (at 20 NS) for brainstem and spinal cord are 46.7 Gy and 37.6 Gy, respectively ‐ both of which are below their respective tolerances. Plan monitor units as a function of NS setting shown in Fig. 5(d) has a similar trend as PTV1 target coverage, increasing in value by a factor of 1.5 as NS is increased from 20 to 80, with no considerable changes after that. The actual number of plan segments $\left({\text{NS}}_{\text{actual}}\right)$ created by DMPO increases linearly as a function of NS setting, with a slope=0.942, intercept=3.23, and $R^{2}=0.999$ (plot not shown). Deviations were only seen for very large NS settings, ≥300 where noticeably fewer segments are created.

#### B.2. Minimum Segment Area and Local Minima

**Figure 6:**
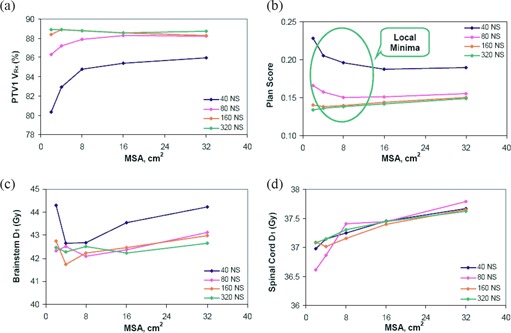
Minimum segment area results. PTV1 target coverage; (b) plan score (Note the unexpected decrease in $V_{\text{Rx}}$ and increase of plan score when $\text{MSA}\leq 8\;{\text{cm}}^{2}$ suggesting presence of local minima); (c) brainstem maximum dose; (d) spinal cord maximum dose as a function of NS.

Figure 6 presents the PTV1 target coverage, plan score, and critical structure maximum doses as a function of MSA setting. Note that the MSA is the minimum segment area allowed for segments in the plan. Therefore, as MSA decreases, the optimization space is larger, and the resulting optimal plan, in principle, should be better. However, this was only seen for larger NS (≥80) and MSA $\left(\geq 8\;{\text{cm}}^{2}\right)$ settings. For example, when the NS setting is held at 80, the plan score presented in Fig. 6(b) increases from 0.150 to 0.155 as the MSA is increased from 8 cm^2^ to 32 cm^2^. Unexpectedly, as the MSA is decreased from 8 cm^2^ to 2 cm^2^, the plan score also increases from 0.150 to 0.166. This becomes worse when NS is reduced to 40.

Similarly, the relationship between target volume coverage and MSA setting resembles that of plan score with MSA setting. As shown in Fig. 6(a), at larger NS settings (≥80), the $V_{\text{Rx}}$ values for PTV1 and PTV2 (not shown) decrease as the MSA setting is reduced from 8 cm^2^ to 2 cm^2^. For smaller NS (≤40), the degradation in plan quality is continuous as the MSA setting is reduced from 32 cm^2^ to 2 cm^2^.

As was previously stated, reducing the MSA to smaller values provides a larger optimization search space. In doing so, the optimization algorithm should find a solution that is similar – if not better – than solutions found for larger MSA settings. However, this was not the case. Instead, decreased plan quality was observed for smaller MSA $\left(<8\;{\text{cm}}^{2}\right)$ settings, suggesting that the DMPO is caught in local minima. This may be due to the use of a gradient‐descent optimization algorithm and increased constraints in the optimization problem (low NS) for smaller MSA settings. Only at 320 NS does the plan score match expectations, steadily increasing as the MSA setting is made larger. This demonstrates that for very high NS there may be fewer constraints on the optimization algorithm, such that it is less susceptible to local minima. A possible explanation for this observed behavior is that during the leaf sequencing procedure in DMPO (Fig. 2), only an initial set of MLC positions are created and then constraints on the MLC are observed (i.e. following the setting of MSA). It is very likely that these positions are not optimal; however, due to the nature of the gradient search algorithm, DMPO only looks for better solutions in the nearby search space. When NS is small, a global minima solution may not be in the nearby regions, and DMPO is caught in the local minima. This may not be an issue when NS is large, where many segments are generated in the leaf sequencing stage and the degree of freedom is much larger.

In Fig. 6(b) the plan score values shown are averaged over all 12 patients for each set of DMPO parameter settings (NS and MSA). To confirm that the observed increases of plan score are of statistical significance, a paired t‐test analysis was performed. For the 40–160 NS curves, plan scores for two MSA settings were compared: one at the MSA setting where the reversal in the curve ends (i.e. $\text{MSA}=2\;{\text{cm}}^{2}$), and one at the MSA setting where the reversal in the curve begins (i.e. for 40 NS, this is at $\text{MSA}=16\;{\text{cm}}^{2}$; for 80–160 NS, this is at $\text{MSA}=8\;{\text{cm}}^{2}$). For the 320 NS, there is no reversal of the curve; therefore plan scores were compared between 2 cm^2^ and 8 cm^2^ MSA. Calculated *p*‐values for 40 and 80 NS were both <0.001, demonstrating that the higher plan scores observed for 2 cm^2^ MSA are statistically significant (i.e. we can confirm that there are local minima for these settings). The result for 160 NS was not meaningful with a calculated *p*‐value of 0.696. For 320 NS, the calculated *p*‐value was 0.023 and, therefore, the lower plan score observed for 2 cm^2^ MSA is also statistically significant. In other words, there are no local minima for NS=320. This analysis supports our suggestion of the susceptibility of the DMPO algorithm to local minima at lower $\left(<8\;{\text{cm}}^{2}\right)$ MSA and NS (<80) settings.

Previously, Wu and Mohan^(29)^ as well as Llacer et al.^(30)^ have investigated effects of local minima on gradient‐based IMRT optimization with dose‐volume constraints for clinical cases. Both works found no clear evidence that local minima prevent gradient‐based optimization methods from finding a good solution. Wu and Mohan speculated that this might be due to the large number of beamlets used in IMRT optimization, which prevent the solution from getting trapped in a local minimum. In aperture‐based optimization, the number of variables for optimization is typically less than the beamlet‐based optimization. The number of variables also depends on the parameter settings such as NS, and is almost proportional to the NS. In addition, aperture‐based optimization must also consider the beam delivery system constraints within the optimization. These constraints effectively reduce the number of independent variables in the optimization. Therefore, both the probability of local minima and possibility of getting caught in local minima for low NS settings are high.

**Figure 7:**
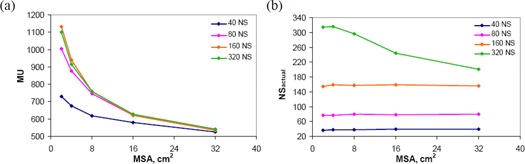
Minimum segment area results. plan MUs; (b): ${\text{NS}}_{\text{actual}}$.

OAR $D_{1}$ values for brainstem and spinal cord are shown in Fig. 6(c) and Fig. 6(d). In both plots, maximum doses were all less than the specified tolerances and there was no specific trend between $D_{1}$ values and DMPO parameters. Figure 7 presents the effect of MSA on the plan monitor units and number of segments in the plan. For all NS settings, plan MUs monotonically decrease with increasing MSA. This agrees with our previous results on the ‘integral MU’ shown in Fig. 4(b). It was previously noted in Fig. 5(d) that for 8 cm^2^ MSA, plan MUs did not significantly change for NS≥80, but Fig. 7(a) demonstrates that, for MSA settings $<8\;{\text{cm}}^{2}$, the relationship between MUs and NS does not saturate as quickly, shown by the increased separation of the 80, 160, and 320 curves. The average number of plan segments $\left({\text{NS}}_{\text{actual}}\right)$ as a function of MSA is shown in Fig. 7(b). For NS≤160, the number of segments in the plan is independent of the MSA setting, ${\text{NS}}_{\text{actual}}=\text{NS}$. However, for NS=320, the average ${\text{NS}}_{\text{actual}}$ decreases from 316 to 204 as the MSA setting is increased. This indicates that as the MSA setting is made larger, there is a limit to the number of segments needed to produce an optimal plan.

**Figure 8:**
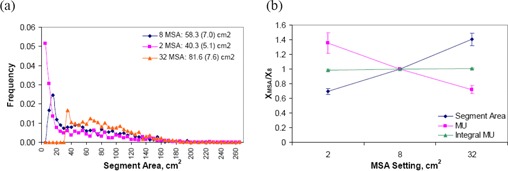
Comparison of DMPO plans with different settings of MSA (2, 8, 32 cm^2^). segment area distributions averaged over all 12 patients. Legend shows mean segment area and standard deviation (shown in parentheses) for each distribution. parameters (mean segment area, plan MU, and ‘integral MU’) at each MSA setting normalized to MSA of 8 cm^2^ averaged over all patients. The error bars represent one standard deviation.

A comparison of the segment area distributions for DMPO plans with varying MSA settings is displayed in Fig. 8(a), with results averaged over all twelve patients. These histograms show that by increasing the MSA setting, the fraction of segments in the plan that have larger areas increases. This subsequently increases the mean segment area for the plan. For an MSA of 2 cm^2^, the mean segment area is 40.3 cm^2^; this increases to 58.3 cm^2^ at 8 cm^2^ MSA, and further to 81.6 cm^2^ at 32 cm^2^ MSA. When an MSA of X cm^2^ is chosen, no segments with area $<X\;{\text{cm}}^{2}$ are found in the resulting plan, demonstrating that the DMPO algorithm performs as intended. Similar to what was shown previously on comparing the segment area distributions for SW and DMPO methods, increasing the MSA leads to more segments of larger area in the plan, which also reduces the total MUs – therefore improving the radiation delivery efficiency. This effect is demonstrated in Fig. 8(b), where the average segment area, plan monitor units, and ‘integral MU’ are plotted for each MSA setting normalized to a MSA of 8 cm^2^. As expected, the ratio of the ‘integral MU’ at each MSA setting is very close to 1. At 2 cm^2^ MSA, the segment area is smaller than that at a MSA of 8 cm^2^ (ratio=0.69) and therefore, in order for the ‘integral MU’ to remain constant between plans, the plan monitor units required is larger (ratio of plan MUs=1.36). Likewise, at 32 cm^2^ MSA, the mean segment area is larger (ratio=1.41) and therefore the plan monitor units required is smaller (ratio=0.72).

### C. Delivery Efficiency

**Figure 9:**
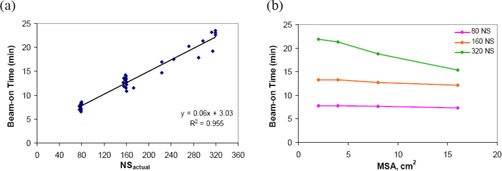
Delivery efficiency results: beam‐on time. measured beam‐on time as a function of the total actual number of segments in the plan; (b) measured beam‐on time as a function of MSA setting. For 320 NS, decreased beam‐on time shown is attributed to decreased ${\text{NS}}_{\text{actual}}$ seen at larger MSA settings.

Results from irradiation time measurements for treatment plans with varying NS and MSA are presented in Fig. 9. In Fig. 9(a), it is clear that the beam‐on time is strongly dependent on the total number of segments in the plan, ${\text{NS}}_{\text{actual}}$, increasing almost linearly (slope=0.06,intercept=3.03, and $R^{2}=0.955)$. The relationship between beam‐on time and MSA is shown in Fig. 9(b) where it is independent of the MSA chosen. At an NS setting of 320, the beam‐on time appears to decrease with increasing MSA, but this decrease in time is attributed to the decrease in ${\text{NS}}_{\text{actual}}$ from 315 to 214 segments (averaged from three patients) as MSA is increased from 2 cm^2^ to 32 cm^2^. With respect to plan monitor units, beam‐on time is only weakly dependent on the plan MUs with a Pearson correlation coefficient of 0.343. These results demonstrate that the estimation of beam‐on time for HN‐IMRT plans on an Elekta linac should be largely based on ${\text{NS}}_{\text{actual}}$ instead of the plan MUs, typically the dominant factor used for predicting treatment times for standard conformal radiation delivery.

For treatment plans with 8 cm^2^ MSA, as the number of segments increases from 78 to 158, the measured beam‐on times increase from ~8 minutes to ~13 minutes. Beam‐on time increases further to ~19 minutes when ${\text{NS}}_{\text{actual}}$ is increased to 264. Plans with ~320 segments were only achieved when the MSA was reduced to 2 cm^2^. At this small MSA setting, plans with an average of 315 segments had a measured beam‐on time of ~22 minutes. Conservative estimates of the total fraction time are ~25, ~30, and ~39 minutes for plans with 78 (NS=80), 158 (NS=160) and 315 (NS=320) segments, respectively, and therefore treatment plans with NS between 80–160 are considered to be practical in a clinical setting.

**Figure 10:**
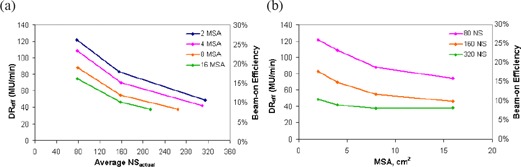
Delivery efficiency results: effective dose‐rate and beam‐on efficiency. average effective dose rate and beam‐on efficiency both as a function of the number of segments in the plan; (b): average effective dose rate and beam‐on efficiency as a function of MSA setting.

The relationships between effective dose rate, ${\text{DR}}_{\text{eff}}$ and beam‐on efficiency with ${\text{NS}}_{\text{actual}}$ and MSA are similar and this is expected based on their definitions (see Eqs. 2 & 3). Therefore, both of these delivery efficiency metrics are displayed on the same plot and shown in Fig. 10 as a function of number of segments (Fig. 10(a) and MSA (Fig. 10(b). For all MSA settings, the average beam‐on efficiency and ${\text{DR}}_{\text{eff}}$ decrease as ${\text{NS}}_{\text{actual}}$ increases. Beam‐on efficiencies for plans with 8 cm^2^ MSA and 79, 158 and 264 segments are 18.5%, 11.4% and 7.9%, respectively. This significant decrease in beam‐on efficiency with increasing ${\text{NS}}_{\text{actual}}$ shows that, for Elekta linacs, caution should be placed when creating plans with a large number of segments because of the high penalty in decreased efficiency. For each NS setting, beam‐on efficiency and ${\text{DR}}_{\text{eff}}$ decrease as MSA increases. For 80 NS plans, as the MSA setting was increased from 2 cm^2^ to 16 cm^2^, the corresponding beam efficiencies decreased from 25.5% to 15.7%. We want to point out that the higher effective dose rates and beam‐on efficiencies at smaller MSA settings are caused by the steady increase in plan monitor units with decreasing MSA setting noted earlier in Fig. 8(a). Considering that irradiation time is independent of MSA, smaller MSA settings should be avoided, in order to minimize the indirect leakage exposure to the patient associated with increased MUs.

### D. Dosimetric accuracy

MapCHECK passing percentage results for DMPO plans with varying MSA and NS revealed that dosimetric accuracy is not affected by the DMPO parameter settings used. For a MSA setting of 2 cm^2^, the passing percentage values for 80, 158 and 312 ${\text{NS}}_{\text{actual}}$ are 89.6±4.4%, 91.3±3.5%, and 91.5±3.4%, respectively. Similarly, for a MSA of 8 cm^2^, the passing percentage values for 80, 157, and 272 ${\text{NS}}_{\text{actual}}$ are 89.3±2.4%, 87.7±4.5%, and 87.0±2.6%, respectively. The passing percentage values are essentially the same for different NS and ${\text{NS}}_{\text{actual}}$ as well as different MSA. Therefore, dosimetric accuracy does not play an important role in choosing optimal parameter settings because even quite complex plans containing many segments with areas as small as 2 cm^2^ can be delivered with accuracy, similar to that of simpler plans (less segments, larger segment areas).

## IV. DISCUSSION

In this study, we first investigated three deliverable optimization methods available in a commercial treatment planning system (TS, SW and DMPO) to determine which deliverable methods are optimal for HN‐IMRT planning on an Elekta linac. Following comparisons with the ideal FO method, we found that our results agree with previous published findings: that aperture‐based algorithms are superior to other methods because of their ability to reduce plan MUs without loss of plan quality. In fact, we believe that not only is DMPO very suitable for HN‐IMRT planning on Elekta linacs, but is in fact needed in order to achieve the most optimal plans. This is shown by the fact that the plan quality of DMPO plans was not just comparable to other methods, but significantly improved with a composite plan score that is ~2.3 times lower than TS plans and ~1.6 times times lower than SW plans. A majority of published studies have concluded that aperture‐based optimization is a possible alternative to two‐step optimization with significant advantages. However, there are very few results that have demonstrated the necessity of using aperture‐based optimization as presented here. This may be attributed to the limited number of studies using Elekta linacs, whose strict beam delivery system constraints create additional challenges for IMRT optimization.

An additional benefit to DMPO noted in this study is that DMPO plan quality results have the lowest standard deviations of any of the deliverable optimization methods. Therefore, with DMPO we can determine a ‘class solution’ of parameters that can be applied to produce consistent results for a variety of patients, regardless of differences in tumor sizes, locations, and surrounding anatomy. This can have a significant impact on clinical operation because the complex treatment planning process is streamlined, treatment time and throughput can be estimated accurately, and quality assurance task can be significantly simplified.

Another interesting finding of this study is that, in addition to the number of segments, there are other factors that have an influence on the total MUs. A comparison of segment area distributions for different optimization methods as well as for varying MSA settings established that there is a relationship between total MUs and segment area, which can be explained by a newly proposed concept called ‘integral MU’. Our results demonstrated that, despite differences in planning methods and parameters, the quantity ‘integral MU’ remains constant while segment area and total MU vary. Therefore, the reduction in plan MUs for DMPO plans presented in this work are not the result of less total plan segments but, instead, are due to the DMPO algorithm selectively increasing the area of plan segments to yield a more delivery efficient plan.

Even though DMPO has been available for a few years and many clinics are using it routinely for planning, no studies have been reported on the limitations of this algorithm. We found that for low NS and MSA settings, DMPO may get caught in local minima, producing a plan that is sub‐optimal; caution should be used when extreme parameters are chosen. For example, if the planners are concerned about the treatment delivery time, they can choose a low NS setting. Additionally, they may also set a low threshold of segment area (i.e. MSA setting is also low), hoping to get the best results available from DMPO. While the planners' intentions are good, the results may not be optimal and may be unexpected.

These results may be due to the use of a gradient descent method for optimization, an algorithm type which generally is fast but not sophisticated enough to overcome local minima.^(31)^ This is in contrast to the simulated annealing algorithms incorporated into the in‐house built direct aperture optimization (DAO) used in many published works.^(8)^ Simulated annealing algorithms are, in principle, more robust and less susceptible to local minima. However for sensible use in the clinic, the need to provide a solution within a feasible length of time is generally of higher priority over the generation of a truly optimal dose distribution, making the use of gradient descent optimization algorithms more practical.^(31)^ This unexpected insight underscores the importance of systematic studies like this one for a majority of clinics where commercial software is used instead of research software, and guidance of proper use is much needed.

The significantly increased treatment times associated with complex IMRT plans are of major concern to many clinicians and patients.^(12)^ Based on experience with standard conformal radiation delivery, MUs are assumed to have considerable influence on the overall treatment time. However, our measurements showed HN‐IMRT treatment delivery time has a predominantly linear relationship with the number of plan segments $\left({\text{NS}}_{\text{actual}}\right)$, and is only weakly dependent on plan MUs. This may be specific to the IMRT delivery on an Elekta linac and not applicable to other types of linacs. Protracted delivery times reduce the biological efficacy of radiotherapy by allowing for cell repair, adversely impacting tumor cell killing.^(12)^ ^,^ ^(32)^ Thus, in order to deliver truly optimal patient treatments, this dependence of delivery time on the NS setting should be kept in mind during IMRT planning, instead of focusing on the plan quality alone. In terms of clinical operation, for standard fraction radiotherapy (1.8–2.0 Gy per fraction), total treatment time per fraction exceeding 30 minutes is generally unacceptable. The beam‐on time should be comparable to the time needed for other purposes, such as patient setup, gantry rotation, etc. Therefore, a good rule of thumb is that the beam‐on time should be <½ of the total treatment time (i.e. <15 minutes), and this can be used to define the proper number of segments needed for IMRT planning.

Contrary to the common belief that small segments are the culprit for dosimetric discrepancies between dose calculations and patient QA measurements, our study found this not to be the case. Analysis of MapCHECK measurements shows that dosimetric accuracy is independent of both NS and MSA parameters, and that even plans with the smallest segment area setting (2 cm^2^) can achieve equivalent QA results. These findings may be attributed to careful commissioning of the linac, as well as accurate modeling of small fields in the treatment planning system for IMRT delivery.

## V. CONCLUSIONS

We have compared several optimization methods in the Pinnacle^3^ planning system for HN‐IMRT planning on Elekta linacs. We also introduced and tested the new concept of ‘integral MU’ to explain the relationship between MU and segment area for comparable plans. The superior quality and consistent results of the DMPO treatment plans makes DMPO an appropriate tool to develop a ‘class solution’ suitable for most patients. This directly benefits clinical operation, as treatment planning can be simplified, and treatment time and patient throughput can be predicted accurately. However, the suggested vulnerability of the DMPO algorithm to local minima prompts cautious use of extreme parameters during planning, and emphasizes the need for studies like this one to systematically evaluate the effect of parameters in any new planning techniques. The findings of this work may provide clinical guidance on the use of similar commercial software and delivery equipment. The optimal set of parameters should be chosen to balance plan quality, delivery efficiency, and accuracy. For the objectives acceptable in our clinic, the NS settings between 80–160 are optimal; NS<80 results in unacceptable plan quality and NS>160 results in impractical irradiation times, which are nearly proportional to the number of actual plan segments and offer no significant plan improvement. From this study, we also found that larger MSA settings $\left(\geq 8\;{\text{cm}}^{2}\right)$ are optimal due to the susceptibility of the DMPO algorithm to local minima and the increased MUs at small MSA. Although NS=160 improves plan quality slightly, it also lengthens the treatment delivery time (by ~5 minutes) and therefore our recommended settings for HN‐IMRT on Elekta linacs are NS=80 and $\text{MSA}=8\;{\text{cm}}^{2}$. Furthermore, we want to emphasize that these results are also specific to the SIB‐IMRT treatment technique used. For clinics that use IMRT to treat upper neck only, optimal parameters may be different.
